# Metabolic changes in the developing sugarcane culm associated with high yield and early high sugar content

**DOI:** 10.1002/pld3.276

**Published:** 2020-11-11

**Authors:** Virginie Perlo, Frederik C. Botha, Agnelo Furtado, Katrina Hodgson‐Kratky, Robert J. Henry

**Affiliations:** ^1^ Queensland Alliance for Agriculture and Food Innovation University of Queensland Brisbane QLD Australia

**Keywords:** development, fiber, metabolome, *Saccharum*, sugar, sugarcane

## Abstract

Sugarcane, with its exceptional biomass and sugar yield, has a high potential for the production of bioenergy, biomaterials, and high‐value products. Currently, the link between metabolic changes in the developing internodes in sugarcane and final yield and sugar characteristics is not well understood. In order to investigate these correlations, 1,440 internodes were collected and combined to generate a set of 360 samples across 24 sugarcane cultivars at five different developmental stages. A combination of metabolome profiling and trait co‐expression analyses were conducted to reveal the interaction between the metabolome and essential agronomic traits, including Brix (total sugar), polarity (sucrose content), purity (sucrose purity), commercially extractable sucrose, fiber, and tons of cane per hectare (TCH). Metabolomic analysis revealed significant differences in metabolic patterns mainly correlated with developmental stage. Hierarchical clustering of genotypes and traits revealed clear partitioning of groups of early‐, mid‐ and late‐season sugar content, with secondary segregation by the yield trait, TCH, and fiber content. The study identified co‐expression and specific metabolites associated with metabolic pathways correlated with Brix and fiber content. Knowledge of the correlation between co‐expressed metabolites and diverse agronomic traits will allow more deliberate selection of genotypes for early or late sugar development and fiber content and biomass yield.

## 1 INTRODUCTION

Sugarcane is a major crop grown in the tropical and subtropical regions of the world, producing thelargest biomass of any other crop at over 1.9 billion tons in 2018 (FAOSTAT, 2020). Sugarcane has a significant advantage over most other potential biomass crops because of its long history of industry research and development and the existing infrastructure that is currently used for traditional sugar production (Botha & Moore, 2013). Life cycle analyses indicate that sugarcane would be highly competitive with other crops as a preferred feedstock for a biomass‐based industry (Renouf et al., 2008; Tilman et al., 2006).

In addition to the extensive growing and processing technologies, the sugarcane production systems are underpinned by extensive breeding, research, and development programs producing new sugarcane varieties improved for yield and to overcome problems associated with existing varieties.

The sugarcane industry as it stands today evolved for maximizing the production of sucrose and obtaining economic benefit from the by‐products molasses and bagasse (Botha & Moore, 2013). However, sugarcane has the potential to generate large‐scale recyclable bioplastic, bioenergy, and valuable by‐product, such as dietary supplements, sweeteners, and phytochemicals.

Although it is widely accepted that significant genetic diversity for sucrose and fiber content exists in sugarcane germplasm, modern varieties suggest that achieving high biomass production with both high sucrose and fiber content is unlikely (Jackson, 2005).

Both genetic selection and the production systems of most sugarcane breeding programs are aimed at maximizing sucrose content rather than biomass yield. Reorientation of the production system to harness the total aboveground biomass and growth for maximum biomass has the potential to almost double bioenergy yield (Alexander, 1988). However, there is an increasing realization that in addition to a changed management system gains can be made by improved selection for higher fiber and higher biomass varieties (Chong & O'Shea, 2012).

Despite its high‐yielding nature, sugarcane's experimental maximum yield (212 t/ha) remains lower than those calculated from crop models (Waclawovsky et al., 2010).

As both the genetic selection and production systems are focused on only an end‐of‐season measurement of sucrose yield, there is little information regarding in season fluctuation of the three major traits, biomass, sucrose and fiber, and the metabolic processes that underpin the final yield. Linking early in‐season metabolic changes (metabolome, transcriptome) to the final yield could greatly enhance current attempts to improve and diversify the sugarcane production system. In addition, it would provide significant opportunity to avoid the onset of RGP, and information regarding other potential traits of interest.

In this study, 24 genotypes, representing the selection progress of almost 70 years in the Australian breeding program, were analyzed. The approach was to study metabolome changes in internodes at different developmental stages and attempt to provide a correlation with final sucrose and fiber content.

For this purpose, we determined Brix, polarity, purity, and commercial cane sugar (CCS) that are commonly used to assess and improve quality and value of sucrose. Total cane yield per hectare (TCH) linked to fiber content has been widely used to define and select competitive cultivars for sugar yield. Breeding programs with a controlled and optimum combination of these parameters may lead to enhancements in high‐quality sugar yield or other commercial requirements. For these reasons, associations between these components have been widely described and are still studied intensively (Hoang et al., 2017; Legendre, 1970).

More precisely, Brix or degrees Brix refers to the measurement of total sugar without differentiation between reducing sugars and sucrose in juices. Brix measurement approximates the percentage of sugar by mass. Polarity or apparent sucrose content (Silva et al., 2017) is a major trait to determine the price of sugarcane, reflecting the sucrose content of the sugar (Xiao et al., 2017). Purity is another important index of quality that is utilized to differentiate pure sucrose crystals to natural impurities, such as other sugars (glucose, fructose) and inorganic compounds (ash, colorant). Commercially extractable sucrose represents the sugar content of cane, determined from Brix, polarity, and fiber content of the cane (Mat et al., 2014).

In this project, weighted gene co‐expression network analysis (WGCNA) was used to explore dynamic co‐expressed metabolites and highlight key metabolites linked to Brix, polarity, purity, CCS, fiber, and TCH across different internodes during the development. This analysis method showed that most metabolites from similar biological classes, such as sugar acid, amino acids, steroids, or sugars were co‐expressed together. The results demonstrated that metabolite expression was highly dependent on developmental stages. Profiles based on hierarchy cluster analysis of metabolites expression were maintained across the cultivar for each of the developmental stages.

Sugarcane is known to accumulate sucrose during development (Lingle and Thomson, 2012). Sucrose is a soluble disaccharide initially generated by photosynthesis. This sugar is composed of two molecules––glucose and fructose. During maturation, sucrose is stored in parenchyma cells (Hawker, 1965). The cleavage of this disaccharide dissociates into two molecules. By phosphorylation, the glucose produces glucose‐6‐phosphate, which is a precursor of starch—a polysaccharide containing the glucose units that can be utilized. Fructose and uridine diphosphate)‐glucose (UDP) are also inserted into structural biomass, such as cellulose, hemicelluloses, and lignin (Gibeaut 2000; Wang et al., 2013; Patrick et al., 2013).

Transfer of sugar from distinct tissues, cells, or subcellular compartments requires a set of metabolite transporters. ATP‐binding cassette (ABC) transporters constitute this large class of proteins that are able to allocate assimilated carbon through the plant. Sucrose transporters (SUT/SUC), tonoplast monosaccharide transporter (TMT), sugars will eventually be exported transporter (SWEET), or monosaccharide transporters (MSTs) have been identified as highly expressed sugar transporters in sugarcane with an essential role in carbon partitioning and for crop yield (Chen et al., 2015; Ludewig & Flügge, 2013; Hu et al., 2017, Casu et al., 2003).

The synthesis of amino sugar and nucleotide sugar involves a large family of transporters and transferases. Sugar nucleotides are the substrates to produce major polysaccharides such as glycogen, starch, and cellulose. Galactose is one of the precursors of the amino sugar and nucleotide sugar metabolism.

The objective of this research was to reveal the correlation between essential agronomic traits, such as early‐, mid‐, late‐season sugar and TCH with metabolites and KEGG pathways associated at different developmental stages.

The aim of the study was to display essential metabolites that may be used as biomarkers and give information on the mechanism of carbon fixation associated with sugar release and accumulation as lignocellulosic biomass (Mizrachi et al., 2017; Collucci et al., 2019).

## 2 RESULTS

### 2.1 Traits

**FIGURE 1:**
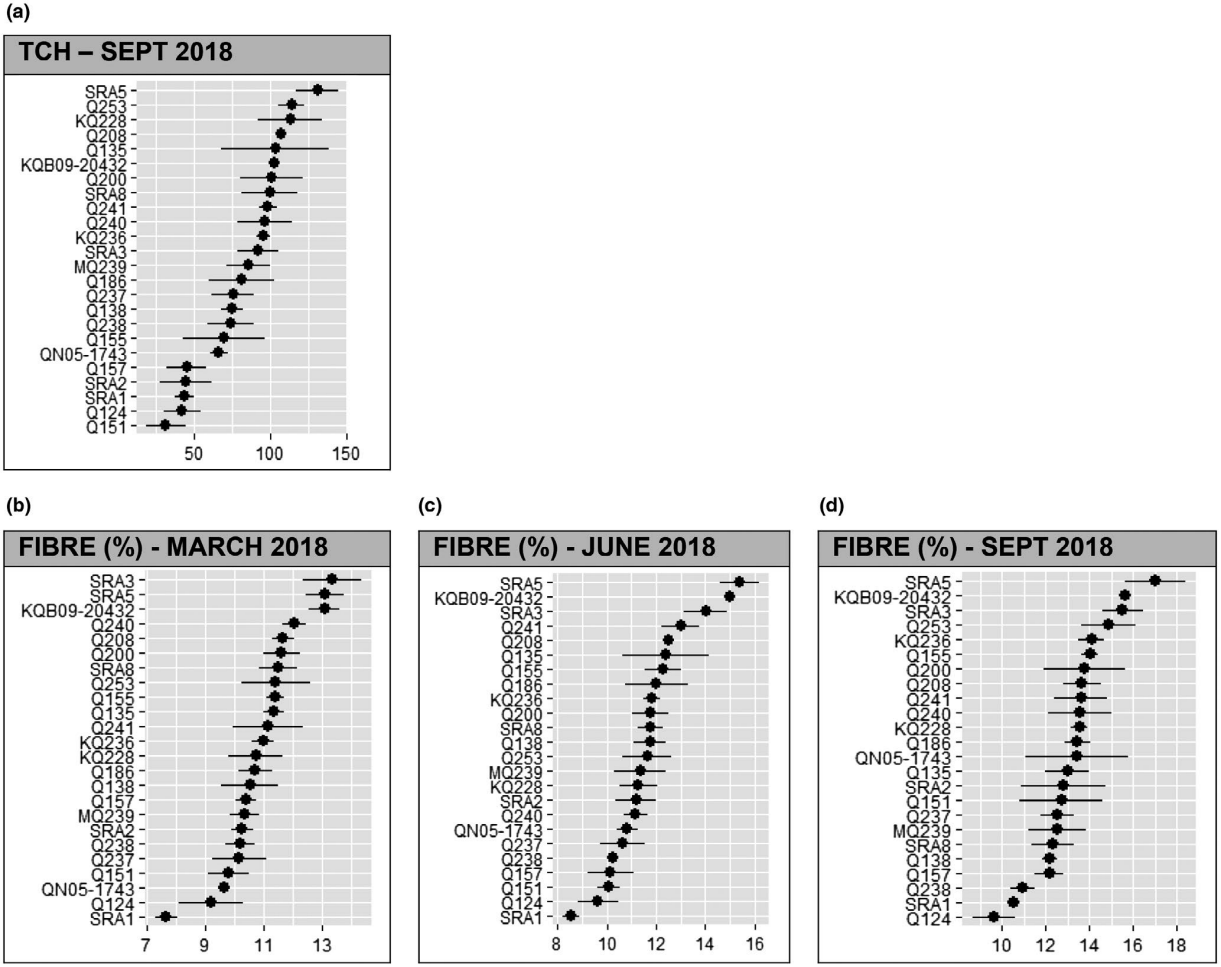
(a) Tons of cane per hectare (TCH) measured in September of 24 commercialized sugarcane cultivars. Fiber content (%) measured in (b) early season (March), (c) mid‐season (June), and (d) late season (September). Means (*n* = 3) presented ± *SD*. The *n* = 3 being the number of samples that were used to calculate the mean for each genotype

Significant segregation for yield (TCH) was identified among the 24 genotypes used in this study (Figure 1a). Three groups of varieties with high, intermediate, and low yield were evident. The first group included around 10 varieties dominated by SRA5, Q253, KQ228, Q208, Q135, and KQB09‐20432 with more than 100 tons of sugarcane per hectare.

Fiber content described for the three seasons–early (Figure 1b), mid (Figure 1c), and late (Figure 1d)—showed shifts of the varieties in the ranking. During the early stage, three clusters were clearly apparent with SRA3, SRA5, and KQB09‐20432 as varieties with high fiber content and SRA1 with distinctly low fiber content. Despite variations in ranking between the varieties, during the different seasons, the three genotypes (SRA3, SRA5, and KQB09‐20432) with the highest fiber content stayed at the top of the ranking and SRA1 together with Q124 consistently rated lowest.

**FIGURE 2:**
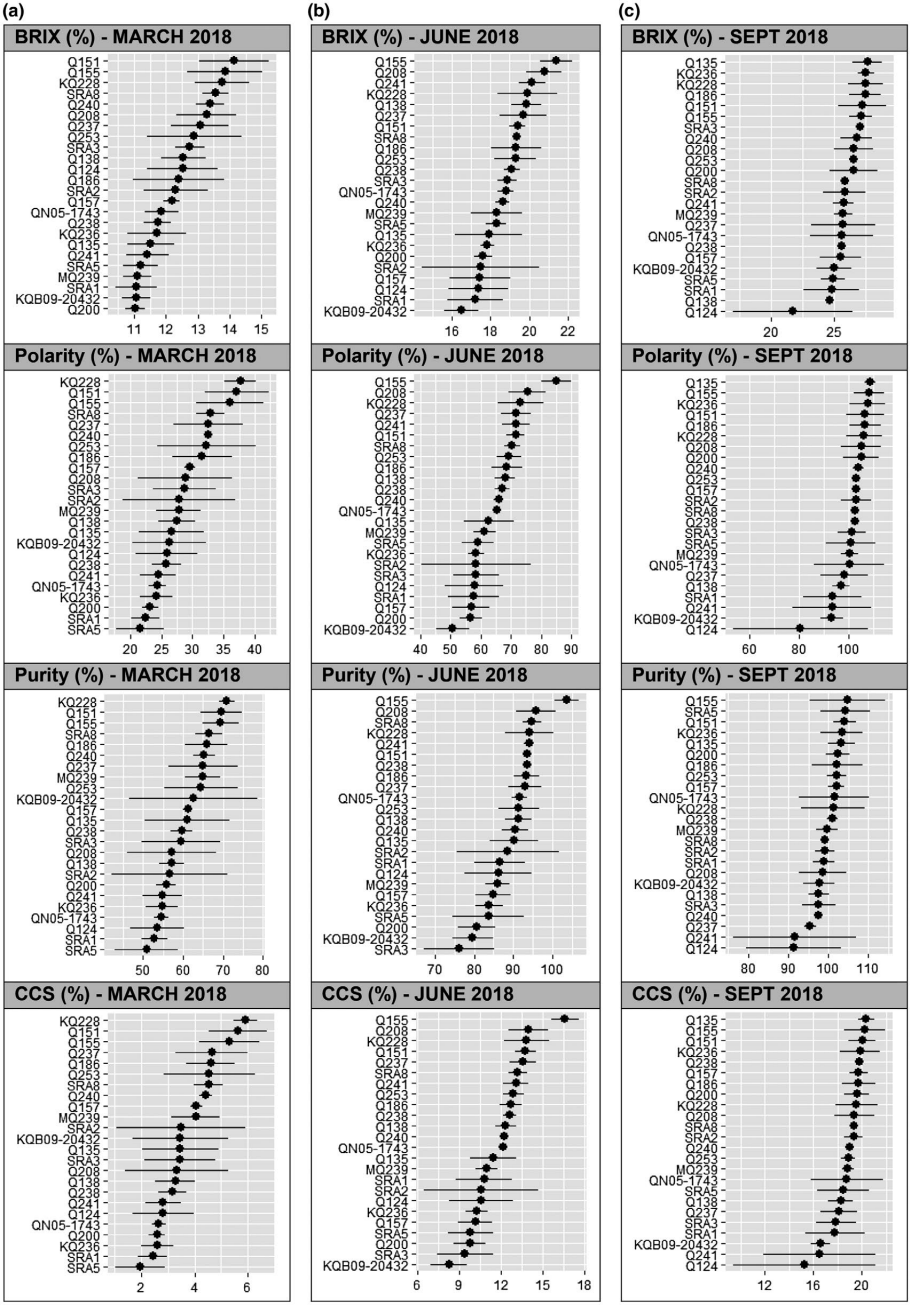
Brix, commercial cane sugar (CCS), polarity, purity percentage measured in (a) early, (b) mid, and (c) late season for 22 commercialized and two unreleased experimental sugarcane cultivars. Means (*n* = 3) presented ± *SD*. The *n* = 3 being the number of samples that were used to calculate the mean for each genotype

Brix degree varied from around 11% to 27.5% across the genotypes and seasons (Figure 2). Varieties were not clearly clustered during each of the three seasons, early (Figure 2a), mid (Figure 2b), and late (Figure 2c), rather they presented a regular gradient of Brix level. Large variations in rankings between the genotypes were displayed across these periods. Varieties such as Q155, Q151, and KQ228 were distinctly characterised by early‐season sugar content while Q135, KQ236, Q186, and Q200 were late‐season sugar content varieties. This distinction took into consideration the Brix content in the early, mid, and late seasons.

Among the 24 genotypes, polarity measurements were displayed across the early, mid, and late seasons (Figure 2). In the early season, two distinct groups with low (SRA5 and SR1) and high (KQ228, Q151 and Q155) polarity content were shown (Figure 2a). The ranking changed significantly during the different seasons. During the mid‐season, Q155 was distinctively separated from the other genotypes with the highest polarity measurement. During the late season, almost all the cultivars reached 100% polarity.

Purity in the early season showed a high similarity of ranking across the 24 genotypes with polarity revealing a strong correlation. Two segregated groups with high and low polarity and purity were represented, respectively, by KQ228, Q151, and Q155, and by SR5 and SR1. During the mid‐season, the variety Q155 had a significantly higher purity than the other genotypes. During the late season, Q155 maintained the highest purity reaching 100% (Figure 2c).

With a similar ranking between the different varieties, these results highlight a very strong positive correlation between CCS and polarity during the three seasons. This correlation between CCS and polarity was also linked to a positive association with purity and Brix content. The greater similarity of ranking of genotypes in early and mid‐season between Brix, CCS, polarity, and purity revealed their highest correlation at these periods.

TCH and fiber were highly positively correlated and interestingly TCH seemed negatively correlated with CCS, Brix, purity, and polarity of the early season.

### 2.2 Traits correlation

**FIGURE 3:**
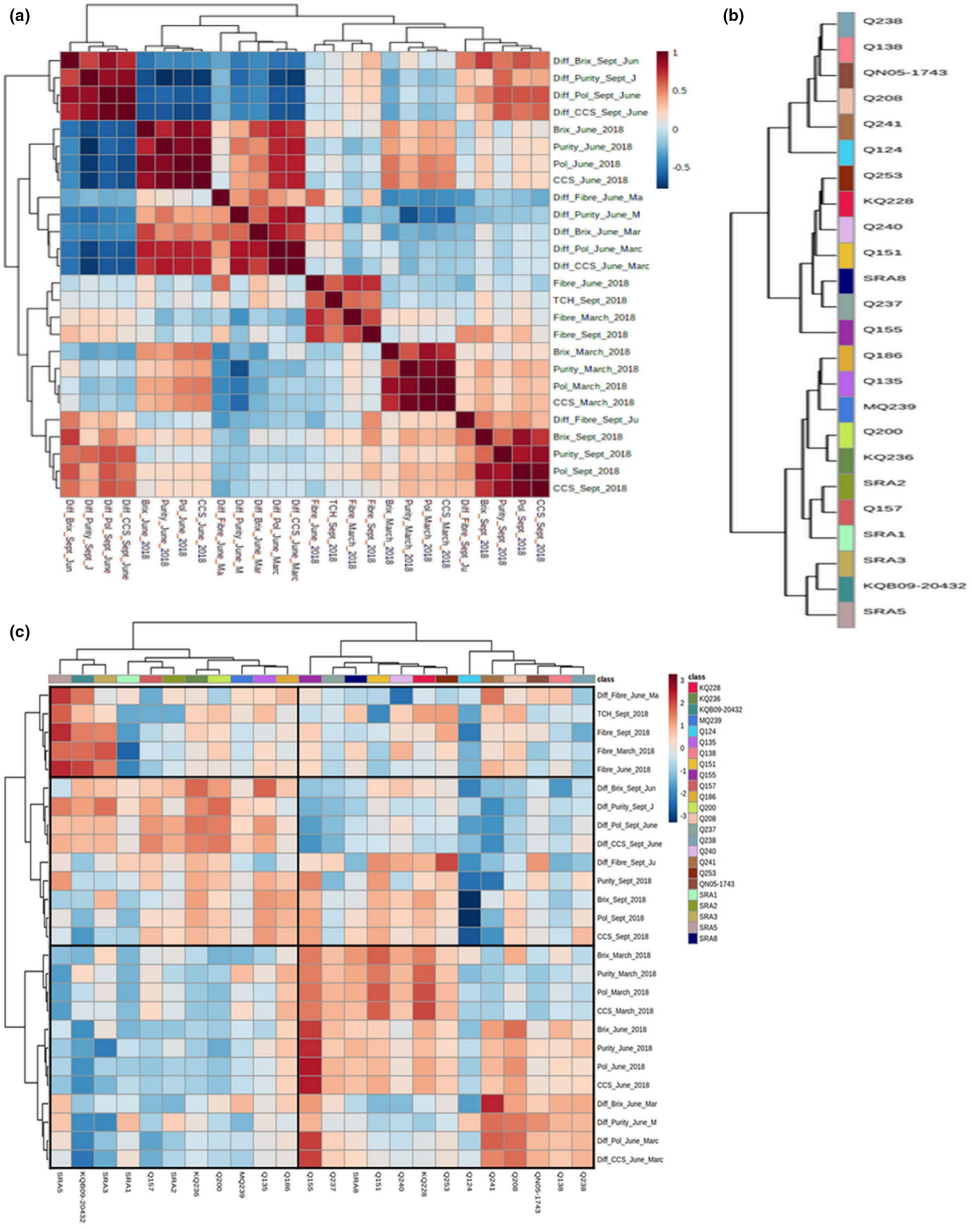
(a) Pearson correlation heatmap of the index quality traits. (b) Hierarchical dendrogram illustrating traits correlation across the 24 varieties, from two‐way hierarchical heatmap. (c) Two‐way hierarchical cluster heatmap, Euclidean distance ward.D clustering algorithm, based on 24 cultivars and agronomic traits. On the top genotypes clustered by traits, revealing two principal clusters, the late‐season sugar content cultivars on the left and the early‐ and mid‐season sugar content cultivars on the right

A heatmap based on Pearson correlation coefficients (Figure 3a) indicated a strong positive correlation between the TCH and fiber content at early (March), mid (June) and late seasons (September). Brix, purity, polarity, and CCS were, respectively, positively correlated for the three seasons (early, mid, and late).

The difference in Brix content between mid and late season and between late and mid‐season was calculated to assess the degree of variation between these periods. Similar difference was obtained for polarity, purity, CCS, and fiber content.

This heatmap revealed a strong positive correlation between the mid‐season sugar content and the difference in sugar content between mid‐ and early‐season. High sugar content in mid‐season was also associated with low accumulation of sugar in late‐season (between June and September). The heatmap also presented a slight negative correlation between TCH and CCS degree.

A post hoc one‐way analysis of variance (ANOVA) (Table S1) identified that the two traits with the most significant segregation between the genotypes were fiber and TCH, followed by early‐ and mid‐season purity and Brix.

Hierarchical cluster analysis (HCA) based on Euclidean distance and Ward's clustering algorithm among agrocommercial traits revealed two main classes of genotypes (Figure 3b,c).

The first group consisted of the genotypes with a high accumulation of sugar during the late season associated with a low sugar content during the early‐ and mid‐seasons. This cluster represented the late‐season sugar content varieties. Genotypes included in this group were divided into three subsets, high, low, and medium TCH and fiber content, respectively, with SRA5, KQB09‐20432, SRA3, with SRA1, Q157, SRA2, KQ236, and finally with Q200, MQ239, Q135, and Q186.

The second group was defined with a low accumulation of sugar during the late season. This group was further divided into three groups, the first one, represented by the early‐season sugar content varieties, including Q155, SRA8, Q237, Q151, Q240, KQ228, and Q253. Q155 was defined by a high sugar content at the early season followed by an additional strong accumulation of sugar in the mid‐season. The second group was characterized by genotypes with high sugar content in the mid‐season and with a high accumulation of sugar in the mid season––including Q241, Q208, QN05‐1743, Q138, and Q238. The third group was distinguished from the others with a significant low sugar content in all seasons, and low TCH and fiber content represented by Q124.

### 2.3 Developmental stages

**FIGURE 4:**
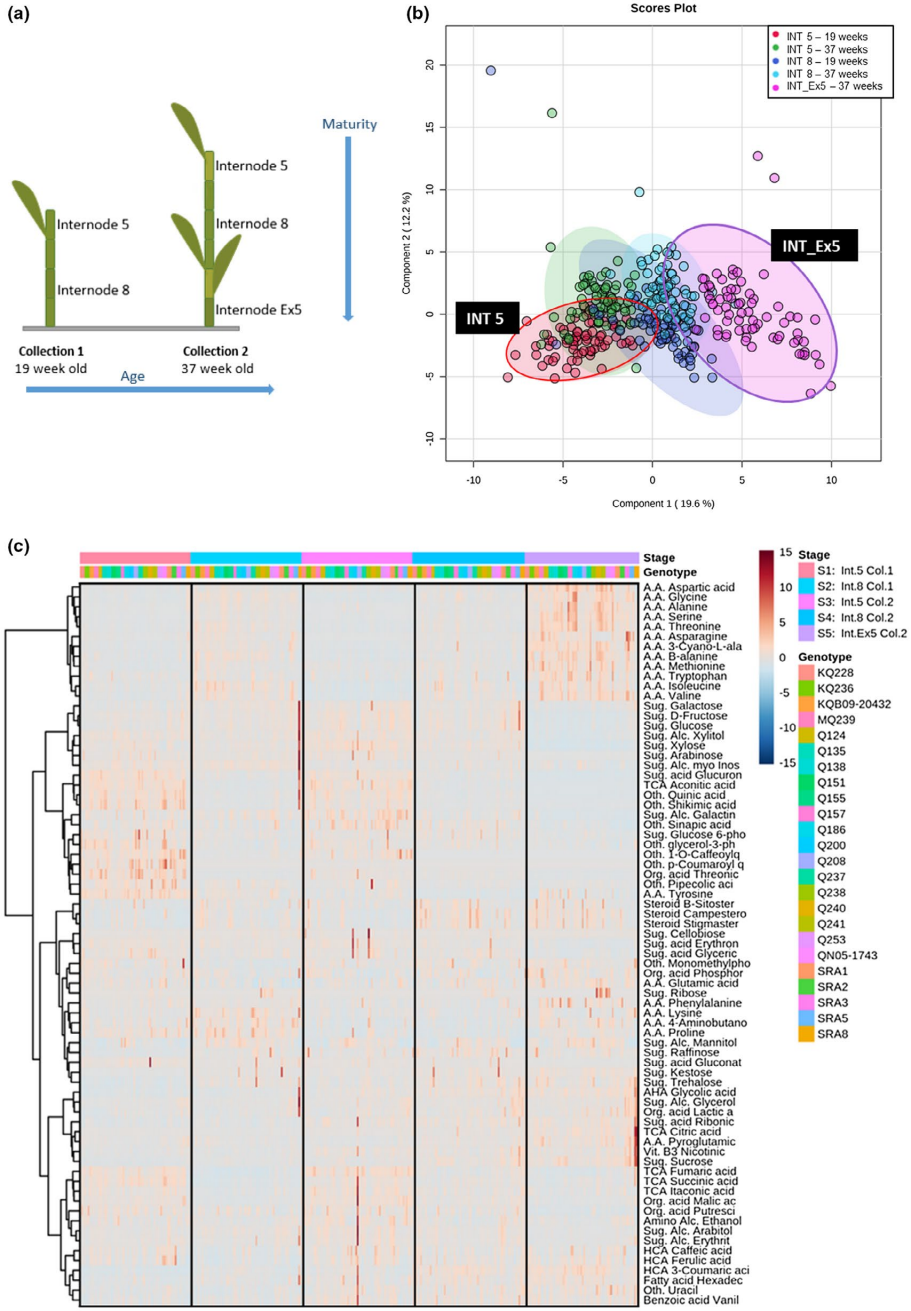
(a) Schematic view of the different stages of development. (b) PLS‐DA from metabolomic data at five different stages, collection 1 (19 weeks) internode 5 and 8, collection 2 (37 weeks) internode 5, 8, and Ex‐5. Scores plot generated using MetaboAnalyst. (c) Heatmap profiling 74 metabolites normalized relative concentrations with samples and metabolites hierarchical clustering

Sugarcane samples represented five stages of development. Internodes 5 and 8 after 19 old and internodes 5, 8 and “INT_Ex5”, 37 weeks old. INT_Ex5 represented the internode 5 tagged during the first period but collected in the second period (Figure 4a).

One‐way ANOVA analysis performed to calculate the variations among the 74 metabolites revealed that 72 metabolites differed significantly (*p* < .05) between genotypes at different stages of development, 55 at an immature stage (internodes INT5), and 60 at a mature stage (INT_Ex5) among the 24 genotypes (Figure S1). Details of the significance and variation in the biochemical measurements of three sugars, sucrose, glucose, and fructose described a highly significant difference in their content across the genotypes and developmental stages (all *p* < .05). This results also showed enrichment for the early sucrose accumulation genotypes and the prevailing sucrose content. This would imply that sucrose accumulation in the early‐ripening genotypes starts very early in the cropping cycle. Simple sucrose test in a young internode may be used as a marker that is not linked to destructive sampling and can be conducted in‐field. Simultaneously, these results described a decrease in fructose and d‐glucose content between the immature stage and the oldest mature age (Figure S2).

Partial least squares discriminant analysis (PLS‐DA) on all 74 metabolites was conducted across the five developmental stages to compare their metabolic compositions. A PLS‐DA score plot exhibited a total variability of 31.8%, illustrating a clear separation of the samples during the five different stages. This cluster analysis revealed that the metabolome or metabolite expressions were apparently different between internode maturity and seasonality. In addition, a PLS‐DA score plot revealed the closest clusters in younger (19 weeks) and older (37 weeks) internodes 4 and between younger and older internodes 8 and finally the cluster linked to the internodes Ex‐5 (INT_Ex5) was clearly more distant from the other groups. This cluster analysis expressed more similarity between internodes with the same position (same maturity), such as internodes 5 at different ages (19 and 37 weeks) than between internodes with similar age but different maturity such as internodes 5 and 8 (Figure 4b).

This separation of metabolic expression during the five different stages was also illustrated by the two‐factor heatmap of normalized relative concentrations. Hierarchical clustering, on the left of the heatmap, revealed that metabolites with similar biochemical properties (such as the amino acids, monosaccharide sugar, steroids, sugar, sugar alcohol, or tricarboxylic acid cycle (TCA) and *p*‐hydroxycinnamic acid (HCA) compounds) were mostly clustered together.

This correlative behavior in metabolite levels suggested co‐expression of metabolites from similar compound classes (Figure 4c).

One of the most significant results was that metabolism characteristics were predominantly correlated with the developmental stage. For this reason, the following correlation analysis with the 24 genotypes was processed for each stage with a focus of the most contrasting stages “INT5” (the youngest) and “INT_Ex5” (the oldest and most mature stage).

### 2.4 Metabolomics–traits correlation across stages and cultivars

The results described above, revealing the significance of developmental stages and the association of metabolites by compound class (Figure 4c), reinforced the potential to analyze the correlation between metabolites and also between genotypes and metabolites across the two‐developmental stages INT5 and INT_Ex5.

**FIGURE 5:**
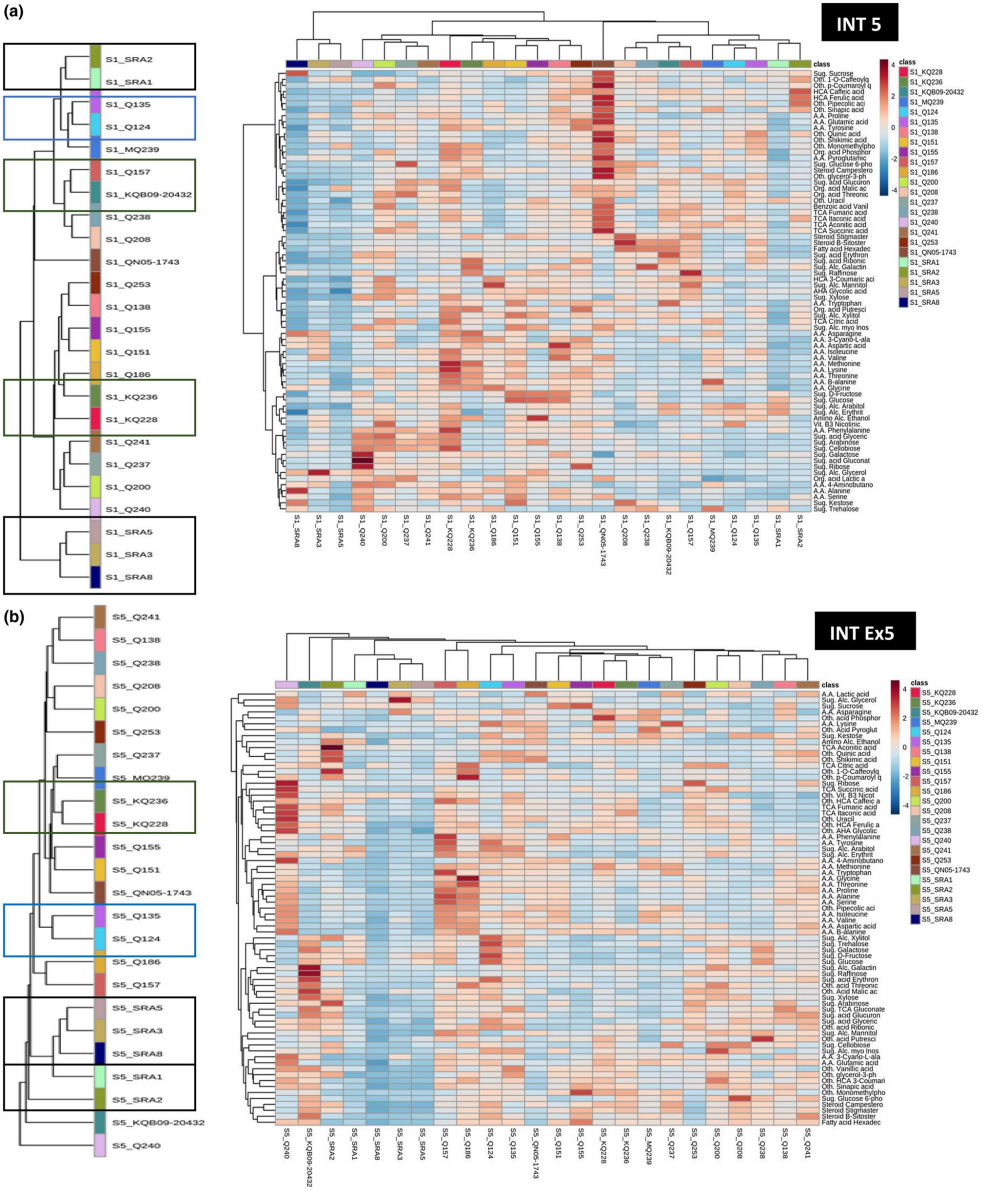
Hierarchical heatmap (a) for INT5 (b) for INT_Ex5, profiling metabolites normalized relative concentrations with samples of 24 varieties. Three samples were used to calculate the mean for each genotype. Clustered of genotypes, horizontal axis and metabolites vertical axis using euclidean distance measure and unweighted pair group method algorithm for clustering. Positive correlation was represented in red and negative in blue. On the left, the detail of the top dendrogram of the 24 varieties have been displayed for a better visibility

The HCA dendrogram of the metabolic expression of the 24 cultivars showed a highly conserved hierarchy between the two stages INT5 and INT_Ex5 (Figure 5a,b). Q135 and Q124 from the same two parents, NCo310 and QN54‐7096 (Table S2), were clustered together for INT5 and INT_Ex5; similarly SRA1, SRA2 and SRA3, SRA5, and SRA8 stayed clustered together. KQ228 and KQ236 and Q157 and KQB09‐20432 also stayed grouped together during these two stages.

The hierarchically clustered heatmap of the 74 metabolites of the 24 genotypes for INT5 (Figure 5a) and INT_Ex5 (Figure 5b) displayed some similar clusters of varieties during the two stages, as SRA8, SRA3 and SRA5 revealed which metabolites were similarly expressed. For instance, this cluster of SRA varieties seemed to be characterized by a low level of all the metabolites except for the sugar alcohol, glycerol, for INT5, and INT_Ex5. Hierarchical clusters of metabolites, on the left of the heatmap described co‐expression of metabolites from the same biochemical class as amino acids, sugars, sugars alcohol, HCA compounds, or steroids.

Metabolite–metabolite correlation (Figure S3) where metabolites were clustered according to their class. For example, amino acids, steroids, fatty acids and sugar acids were respectively positively correlated together. These results also revealed a strong correlation between the majority of chemical components of tricarboxylic acid cycle (TCA cycle), uracil, nicotinic acid (vitamin B3), vanillic acid (benzoic acid), HCA compounds, glycolic acid (AHA), and sinapic acid. Sugar and sugar acids were mostly highly positively correlated. This heatmap described a variation in the correlation coefficient between the two stages, with a generally stronger correlation between metabolites during the youngest stage.

**FIGURE 6:**
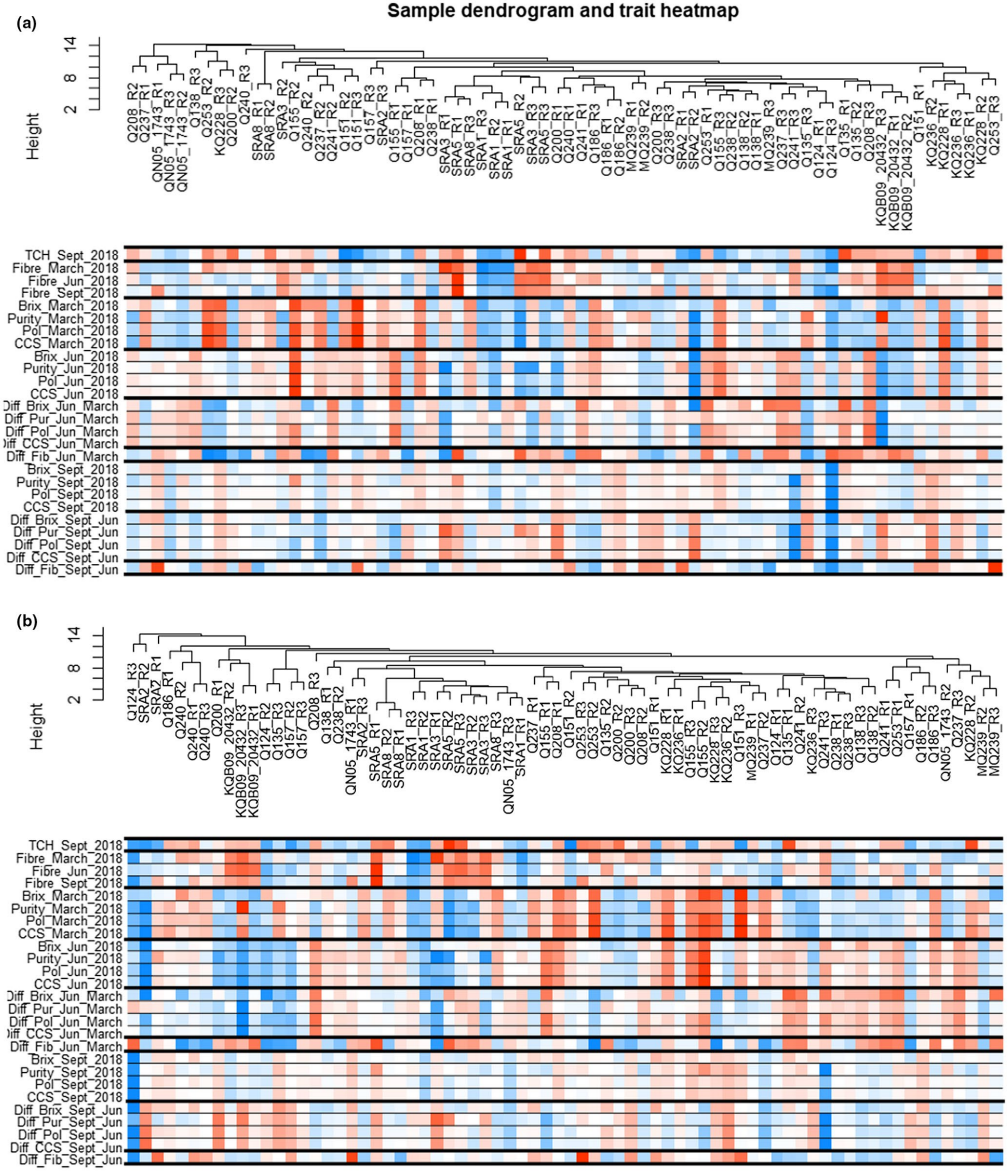
Sample dendrogram and trait heatmap. Traits were the TCH (Tonne of Cane per hectare), the early‐, mid‐, and late‐season fiber, Brix, purity, polarity, commercial cane sugar (CCS) levels, and respective differences of levels between seasons for (a) internode 5, 19 weeks old. (b) Ex‐Internode 5, 37 weeks old. Red color represents positive correlation and blue negative correlation

Further investigation of metabolite correlation was obtained with WGCNA. Hierarchical clustering was performed to produce a hierarchical clustering tree of the metabolites. This dendrogram was correlated with economically essential traits, TCH in September, fiber, Brix, purity, polarity, and CCS in early, mid, and late seasons in the form of a heatmap for INT5 and INT_Ex5 (Figure 6a,b).

This heatmap of correlation with samples and traits illustrated the measurement of metabolites during the different stages of development. The results validated the robustness of the analysis, as the cluster of the high fiber cultivars, SRA5, SRA3, SRA8, Q240, and KQB09‐20432, were strongly maintained for INT5 and INT_Ex5. Early sugar content cultivars, such as Q155, Q151, Q237 and KQ228 were positively correlated with Brix, polarity, purity and CCS in early and mid season (for INT5 and INT_Ex5). Late sugar contents with the highest Brix, polarity, purity, and CCS values in September and the highest sugar accumulation in the late season were more related to the cluster composed by Q186, MQ239, and SRA2 for INT5 and INT_Ex5.

**FIGURE 7:**
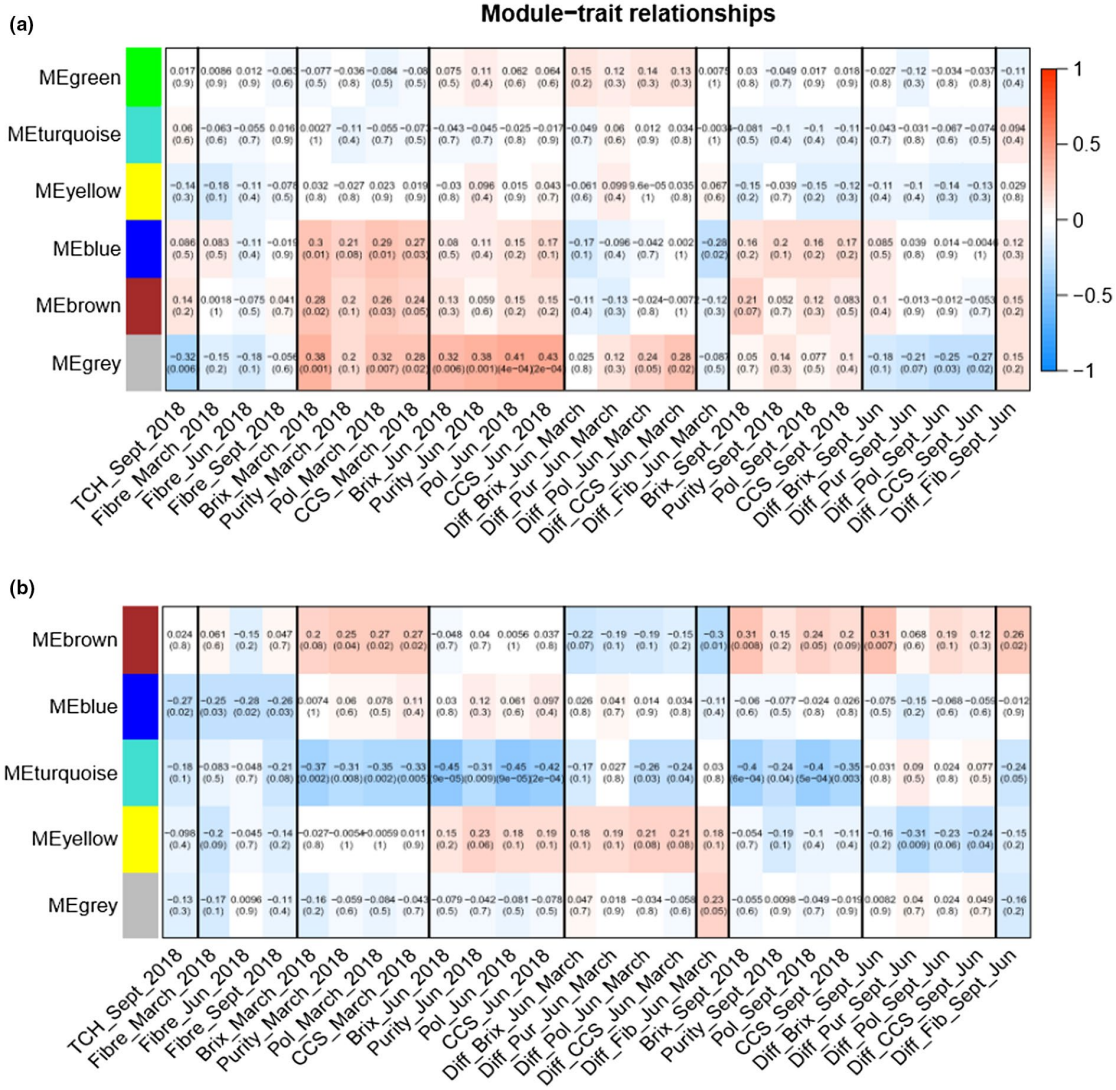
Heatmap of associations between module eigengene (rows) and traits (columns). Each cell contains the module‐trait correlation and in brackets p‐value corresponding. (a) Internode 5, 19 weeks. (b) Ex‐Internode 5, 37 weeks

Module–trait relationships analysis with WGCNA displayed associations of metabolites with the commercial traits, particularly Brix, purity, polarity, CCS, TCH, fiber in early, mid, and late seasons for INT5 and INT_Ex5 (Figure 7).

Each module–trait or module eigengenes (ME) was determined using the average absolute of metabolite significance (MS) measure for all metabolites in a given module correlated to the different traits (Lou et al., 2017).

Early‐ and mid‐season sugar content represented by Brix, purity, polarity, and CCS for March and June displayed a high correlation with the grey module, composed essentially of sugar such as d‐fructose, glucose, kestone, and raffinose, for INT5. Early sugar content was also highly correlated with the brown and blue modules, respectively, associated with the amino acids and the sugars, sugar acids, and sugar alcohols (Figure 7a and Table S3). During the mature age, INT_Ex5, a differentiation of correlation between the sugar traits of early and mid seasons were illustrated, with the brown module represented by metabolites involved in TCA cycle, HCA, uracil, nicotinic acid (Vitamin B3), vanillic acid (benzoic acid), glycolic acid (AHA), sinapic acid, phenylpropanoid, and shikimate highly linked to March sugar traits and the yellow module composed of steroids and fatty acid associated with the mid‐season sugar traits (Figure 7b and Table S3).

The correlation between individual metabolites and traits of interest was defined as the MS. This correlation between the metabolite and the trait Brix content at the mid‐season (Table S4) confirmed a highest positive correlation, with d‐fructose, glucose, kestose (glycan), alanine, isoleucine, and xylitol during the youngest stage INT5 and with the monomethylphosphate, glutamic acid, pyroglutamic acid, and hexadecanoic acid for INT_Ex5. Fiber was positively correlated with galactinol, malic acid, pyroglutamic acid, and raffinose at the stage INT_Ex5 (Table S5).

KEGG pathway enrichment analysis among the 74 metabolites highlighted the change of the relationship between traits and pathways at the different developmental stages. Early sugar content was linked to metabolites from ME Grey, at the stage INT5 related to ABC transporters, starch, and sucrose metabolism and galactose metabolism during the youngest age. At the oldest age (INT_Ex5), a shift of metabolic pathway was apparent with metabolites co‐expressed (ME Yellow) in the steroid biosynthesis and surprisingly negatively correlated with ABC transporters, galactose metabolism, and amino sugar and nucleotide sugar metabolism at this stage.

Similarly, TCH with metabolites from ME Brown, at the stage INT5, was more related to aminoacyl‐tRNA biosynthesis, biosynthesis of amino acids and cyanoamino acid metabolism during the youngest age. Interestingly, at this stage, metabolic pathways linked to TCH were highly negatively correlated to pathways linked to early sugar content, such as ABC transporters, starch, and sucrose metabolism and galactose metabolism. At the oldest age, INT_Ex5, co‐expressed metabolites related to TCH trait (ME Brown) were more related to nicotinate and nicotinamide metabolism, phenylalanine metabolism and phenylpropanoid biosynthesis and negatively correlated to aminoacyl‐tRNA biosynthesis, biosynthesis of amino acids and ABC transporters.

## 3 DISCUSSION AND FUTURE DIRECTION

This research illustrated a distinct positive correlation between the most important agronomic traits Brix, CCS, polarity, and purity and between TCH and fiber.

This study presented a correlation between the metabolomes of the 24 sugarcane varieties and these essential commercial traits. Groups of genotypes of early‐, mid‐, and late‐season sugar content were identified; these groups displayed secondary subsets of high, low, or median TCH and fiber content.

The mid‐season sugar content varieties were characterized with very low accumulation of sugar in early season, a high accumulation of sugar in mid‐season, and minimal or no accumulation in late season. For the late‐season sugar content varieties, similarly rather than a progressive accumulation throughout development, the accumulation was also essentially in late season. This specificity could be a key component for selection for future breeding programs. The results of this study differed from the specification routinely used to characterize the cultivars (Table S2). The reasons for this difference may be due to the correlation network statistic approach used for this research on a large number of samples and cultivars.

The expression of metabolites was predominantly associated with the different developmental stages. Metabolic profiling analysis revealed the preponderant metabolic change during the maturity of the sugarcane. Variations in concentration and co‐expression dynamism of the metabolome explained biochemical mechanisms operating during the different stages. Analysis of dynamic metabolic variations associated with selected phenotypic traits showed potential to be a useful tool to target the optimal harvest time. This study revealed drastic modification of metabolic patterns during development and at the same time, hierarchical clustering of cultivars revealed stability of genotypic relationship across age and maturity.

This research confirmed the potential that metabolites may have to predict ago‐economic traits from the first stage of development. Metabolic markers showed potential for future use in understanding complex polyploid plants such as sugarcane. The study confirms that metabolites, intermediates, or final products of metabolism may be used for predicting and understanding specific traits and were promising as biomarkers (Glassop et al., 2007; Rosato et al., 2018). This might be expected as these metabolites are central to biological process and are the link between genotype and phenotype (Fiehn, 2002) assimilating genetic, transcriptomic, epigenetic, proteomic, and environmental factors (Krumsiek et al., 2016).

High‐throughput metabolic profiling analysis combined with WGCNA proved to be a valuable approach to reveal metabolites and metabolic pathways associated with unique or multiple traits (DiLeo et al., 2011). In this study, co‐expression network analysis revealed key metabolites associated with agroeconomic traits that may be useful to reveal shifts of metabolic pathways associated with these traits, revealing the carbon redistribution during the development.

WGCNA has proved to be an effective tool to detect principal co‐expressed metabolites and key metabolites associated with Brix, polarity, purity, CCS, and fiber associated during the developmental stages and across the 24 genotypes.

In this study, hierarchical cluster analysis applied to metabolic expression reflected the genetic diversity across the 24 cultivars based on parental relationships or targeting breeding improvement. These results suggest hierarchical metabolome dendrogram may be a relevant approach to follow divergence or similarity between genotypes to assist breeding programs.

This information reveals that some traits such as fiber content and late‐season high sugar content may be predicted during the first stage of development. This observation may provide potential for the breeder or grower to be able to estimate sugar content and yield of the selected cultivar at the early stages of cane growth.

Early sugar content was highly positively correlated to monosaccharides such as fructose, glucose at the youngest age and with monomethylphosphate, amino acid as glutamic acid, pyroglutamic acid, and hexadecanoic acid when more mature. These results described complex metabolic changes associated with traits during development, meaning that metabolites were development stage‐trait dependant.

Enrichment pathways analysis revealed the principle role of the ABC transporters. Pathways such as “starch and sucrose metabolism” and “galactose metabolism” revealed to be positively correlated to the early‐ and mid‐season sugar when negatively correlated to TCH in the young internode (INT5). Further investigation of these correlations in future may facilitate developments in genetic engineering with the possibility to increase simultaneously TCH and early‐ or mid‐season sugar content.

Future directions in this area of research should involve the use of a similar approach to associate other multi‐omics datasets with the commercial traits used in this study, for a deeper understanding of the phenotype and breeding value.

The goal of this research was to generate a comprehensible, easy to use, and affordable flexible pipeline to be able to correlate metabolome and agro‐economic traits accessible to breeder and growers. Exploring these joint correlations may be significant for strategic multitrait breeding programs and metabolic engineering.

## 4 EXPERIMENTAL PROCEDURES

### 4.1 Plant materials and field design

A collection of 24 *Saccharum* hybrid cultivars (Table S2) including two unreleased (KQB09‐20432 and QNO5‐1743) was selected for this study. The selected varieties rely on parentage (appearance, harvesting period, and soil preference), seasonal sugar (early, mid, and late sugar), productivity (yield and CCS), germination (speed and reliability), abiotic and biotic stress, and fiber content. Details of these Australian cultivars may be found on free online databases: QCANESelect™ developed by Sugar Research Australia (SRA), CIRAD_TropGENE (Hamelin et al., 2013; Ruiz et al., 2004) developed by French Agricultural Research Centre for International Development (CIRAD), and SugarcaneVariety developed by International Society of Sugarcane Technologists in cooperation in Australia with SRA and the Commonwealth Scientific and Industrial Research Organisation (CSIRO).

A field trial with these 24 genotypes was established on August 29, 2017 at the Sugar Research Burdekin Station in Burdekin, QLD (19°34′08.0″S 147°19′30.7″E). The trial was planted in 6 × 4 Latin square design with three replicates per genotype in order to limit any field variability. All samples were subject to identical environmental growing conditions. Each replicate consisted of 4 m of cane with a 1.52‐m row spacing (Figure S4).

Following soil nutrient testing, the soil was fertilized according to standard industry recommendations (N 160 kg/ha, P kg/ha, K kg/ha and S 20 kg/ha). The sugarcane was furrow irrigated with a 7‐day flood irrigation schedule.

Samples were collected at different time points during the season and final harvest of the plant crop was in September 2018.

### 4.2 Brix, polarity, purity, commercial cane sugar (CCS), and fiber

#### 4.2.1 Sample collection and processing

Six culm samples were taken from the field plots at three time points, May, June, and September 2018. Samples were analyzed with a modified method (Berding, 2010). Culm samples were disintegrated using a Dedini laboratory disintegrator. Subsamples (500 g each) of the disintegrated cane were hydraulically pressed in a Varver (Model CMG 75H‐15) 75‐tonne press. The weights of the resultant plug and expressed juice, *ej* M (g) were recorded.

The plugs were dried at 70°C for 7 days to determine dry weight. Each expressed juice sample was clarified by adding 5% (m/m) OCTAPOL™ (Baddley Chemicals Inc) to the samples. After clarification, the juice samples were subjected to routine analyses for refractometer‐determined soluble solids (Brix ‐ *ej B*) with a S&M ATR Series refractometer, and a Pol with a S&M M100 Polartronic polarimeter (Anon, 1987). Pol % Juice of a solution is the concentration (in g solute per 100 g solution) of a solution of pure sucrose in water having the same optical rotation as the sample at the same temperature. For solutions containing pure sucrose in water, pol % juice is the same as sucrose % juice. For solutions of pure sucrose in water, Brix is equal to the dry substance. Although gases and insoluble solids in suspension may alter the density of a solution, the term Brix refers exclusively to soluble solids.

The Pol% juice and Brix readings were used to calculate CCS which is the payment basis for the purchase of sugarcane from growers. For this purpose, the following formula was applied.

$$\text{CCS}=\frac{3}{2}P\left(1‐\frac{F+5}{100}\right)‐\frac{1}{2}B\left(1‐\frac{F+3}{100}\right)$$

where *P* represents the pol% juice, *B* represents the Brix of juice, and *F* represents the fiber % cane.

### 4.3 Metabolomics

#### 4.3.1 Sample collection and processing

The first collection occurred from January 9, 2018 to January 11, 2018, after 19 weeks of growth and the second occurred from May 15, 2018 to May 17, 2018, after 37 weeks. The samples were collected in the morning hours, from 7:00 a.m. to 11:30 a.m. From four stalks of each genotype, three replicates of internodes 5 and 8 were collected. Counting from the top of the plant, for the purpose of this study it was assumed that internode 5 and 8 were the third and sixth internodes below the first visible dewlap leaf, respectively.

During the second collection, internodes 5, 8, and “Ex5” were collected. Internode “Ex5” corresponded to internode 5 of the first collection which was tagged on stools that were not harvested at this period. In this 19 week period, the crop accumulated 1861°C days (base temperature 11°C) and during the growth period produced 12‐13 new internodes.

Each sample was sliced into small pieces, packaged individually in a labeled bag, and then immediately frozen in liquid nitrogen within approximately 1 min after excision. Samples were briefly stored in dry ice and then transferred to a −80°C freezer for storage.

Each sample was individually pulverized and homogenized under cryogenic conditions using a TissueLyser (Qiagen) for 1.5 min at a frequency of 30 Hz. An equal amount of each sample from the four different stools was pooled for each replicate of each genotype and stored in −80°C freezers. At this stage, there were 360 samples (24 genotypes, 5 internodes, 3 replicates).

#### 4.3.2 Metabolite profiling

GCMS profiling was performed using Metabolomics, University of Melbourne, Australia, as described in Marquardt et al. (2019).

Ten milligrams of freeze‐dried internode powder was dissolved in 2‐ml microcentrifuge tubes with 600 µl of 100% methanol, containing 4% (v/v) internal standards (13C6 Sorbitol and 13C5‐15N Valine) from stock mix. Tubes were shaken at 220g for 15 min at 30°C. They were then centrifuged at 20,000 *g* for 15 min and the supernatant was transferred into new microcentrifuge tubes.

A quantity of 600 µl of Milli‐Q H2O were added to the remaining pellet and vortexed vigorously. The solutions were centrifuged at 20,000 *g* for 15 min. Forty microliters of the supernatant was dried in the speed vacuum for quality control and GC–MS analyses.

### 4.4 Data analysis

A web interface MetaboAnalyst 4.0 (Chong et al., 2018; Xia et al., 2009) was used to integrate and analyze the metabolomics dataset. The multivariate analysis was normalized using a pooled sample from the group with autoscaling (Van Den Berg et al., 2006) given a characteristic symmetric normal distribution, Gaussian, "bell curve" shape.

Enrichment analysis was generated using the module “Enrichment Analysis” of MetaboAnalyst with “Self‐defined metabolite sets.” The customized library was built with KEGG API, interfaced to the KEGG database using the metabolites sets of the 139 pathways of *Sorghum bicolor*.

R packages, ggplot2 (Wickham, 2009), and plyr (Wickham & Hofmann, 2011) were used for exploratory statistical analysis, data organization, and plotting graphs.

The WGCNA R package was used to perform a co‐expression network analysis (Langfelder & Horvath, 2008) and investigate the correlations between metabolites and index quality traits during five different developmental stages.

Initially, hclust and cutreeStatic functions from the WGCNA R package were used to define the appropriate threshold value and to remove outliers. Using hierarchical clustering for outlier detection, seven outliers across the five stages were identified and discarded for all of the analyses.

The function pickSoftThreshold, which performs the analysis of network topology, was used to choose a proper soft‐thresholding power β to which co‐expression similarity was raised to calculate adjacency (Zhang & Horvath, 2005). Based on the data, a default power of 6 was chosen to construct the Topology Overlap Matrix and minimum number of metabolites per module was set at 5.

## CONFLICT OF INTEREST

The authors declare that they have no conflict of competing interest.

## AUTHORS’ CONTRIBUTIONS

F.B., A.F, and R.H. conceived, designed this project, and supervised the research. V.P. drafted the manuscript with input from the other authors and all authors critically revised and approved the final version of the manuscript. A.F. managed the field and laboratory experiments. All authors provided technical support, advice, and performed data analysis.

## Supporting information

Supplementary Material

Table S1‐S6‐Fig S1‐S4
