# Supplementary material for: Metabolic changes in the developing sugarcane culm associated with high yield and early high sugar content
Source: Plant Direct. 2020 Nov 11;4(11):e00276. doi: 10.1002/pld3.276 (PMC7656173; doi:10.1002/pld3.276)
Supplement: Supplementary file 2 — Table S1‐S6‐Fig S1‐S4 [file PLD3-4-e00276-s002.docx]

**Supporting information**

**SUGARCANE METABOLOME ASSOCIATED WITH HIGH BIOMASS YIELD AND EARLY HIGH SUGAR CONTENT.**

Virginie Perlo¹, Frederik C. Botha¹, Agnelo Furtado¹, Katrina Hodgson-Kratky¹ and Robert J. Henry¹

¹Queensland Alliance for Agriculture and Food Innovation, University of Queensland, Brisbane, QLD, 4072, Australia

Corresponding author: Robert Henry, Level 2, Queensland Bioscience Precinct [#80], 306 Carmody Road St Lucia, The University of Queensland, St Lucia QLD 4072.

Email: robert.henry@uq.edu.au,

Tel: +61 7 3346 0552, Fax: +61 7 334 60555

Virginie Perlo

Email: v.perlo@uq.edu.au, [virginie.perlo@gmail.com](mailto:virginie.perlo@gmail.com)

Frederik C. Botha

Email: [f.botha@uq.edu.au](mailto:f.botha@uq.edu.au)

Agnelo Furtado

Email: a.furtado@uq.edu.au

Katrina Hodgson-Kratky

Email: k.hodgsonkratky@uq.edu.au

**Supplemental Figures:**

**Table S1.** Sortable list of traits identified by One-way ANOVA and post-hoc analysis.

**
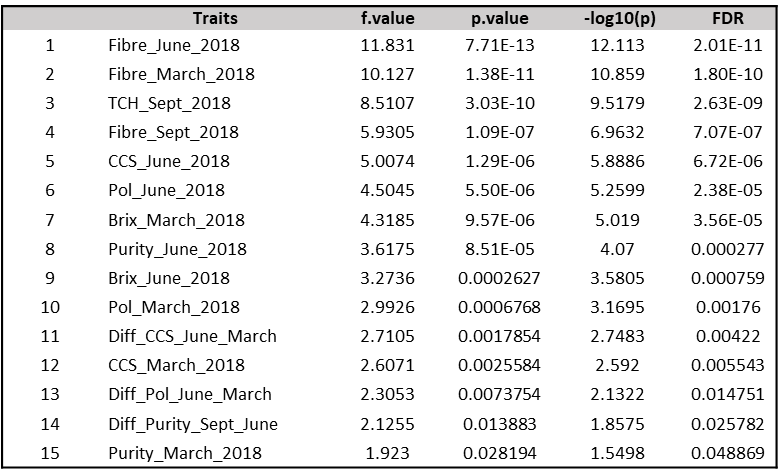
**

**Table S2.** Genetic entities of the 24 genotypes. Colours highlight similar genetics entities.

| **Clone** | **Year of crossing** | **Female** | **Male** | **Year of release** | **Early sugar** | **Mid sugar** | **Late sugar** |
| --- | --- | --- | --- | --- | --- | --- | --- |
| KQ228 | 1993 | QN80-3425 | CP74-2005 | 2006 | Good | Good | Average |
| KQ236 | 1990 | Q96 | POLYCROSS | 2008 | Good | Good | Poor |
| KQB09-20432 | 2009 | KQ228 | QBYN05-10390 | N/A | unknown | unknown | unknown |
| MQ239 | 1992 | Q96 | MQ77-340 | 2009 | Average | Average | Average |
| Q124 | 1960's | NCo310 | QN54-7096 | 1984 | Average | Good | Good |
| Q135 | 1960's | NCo310 | QN54-7096 | 1985 | Poor | Average | Good |
| Q138 | 1970's | QN58-829 | QN66-2008 | 1986 | Poor | Poor | Poor |
| Q151 | ~ 1980 | Q96 | QC66-807 | 1992 | Good | Average | Poor |
| Q155 | ~ 1980 | QC64-386 | Q121 | 1993 | Good | Good | Good |
| Q157 | 1970's | QN58-829 | QN66-2008 | 1993 | Good | Good | Good |
| Q186 | 1980's | Q117 | QN66-2008 | 1999 | Good | Average | Poor |
| Q200 | 1982 | QN63-1700 | QN66-2008 | 2001 | Average | Good | Good |
| Q208 | 1984 | Q135 | QN61-1232 | 2003 | Good | Good | Good |
| Q237 | 1991 | Q120 | CP57-614 | 2008 | Average | Good | Poor |
| Q238 | 1996 | Q138 | Q155 | 2009 | Average | Average | Average |
| Q240 | 1995 | QN81-289 | SP78-3137 | 2009 | Good | Good | Good |
| Q241 | 1991 | Q138 | SP72-4728 | 2009 | Poor | Poor | Average |
| Q253 | 2000 | QN80-3425 | Q209 | 2013 | Poor | Average | Average |
| QN05-1743 | 2004 | QN92-157 | QN90-1820 | N/A | unknown | unknown | unknown |
| SRA1 | 2004 | QN86-2139 | QC90-289 | 2015 | Good | Good | Good |
| SRA2 | 2002 | QS92-206 | QS87-7430 | 2015 | Good | Good | Good |
| SRA3 | 2001 | QN86-2214 | Q200 | 2015 | Poor | Average | Good |
| SRA5 | 2003 | H72-8597 | QN89-109 | 2016 | Poor | Poor | Poor |
| SRA8 | 2000 | QA93-2768 | QA94-6003 | 2016 | Good | Good | Good |

**Table S3.** List of the metabolites associated to eigengenes module (green, turquoise, yellow, blue, brown and grey) of the module-trait relationships heatmap for on the top Internode 5, 19 weeks (INT5) and at the bottom Ex-Internode 5, 37 weeks (INT_Ex5).


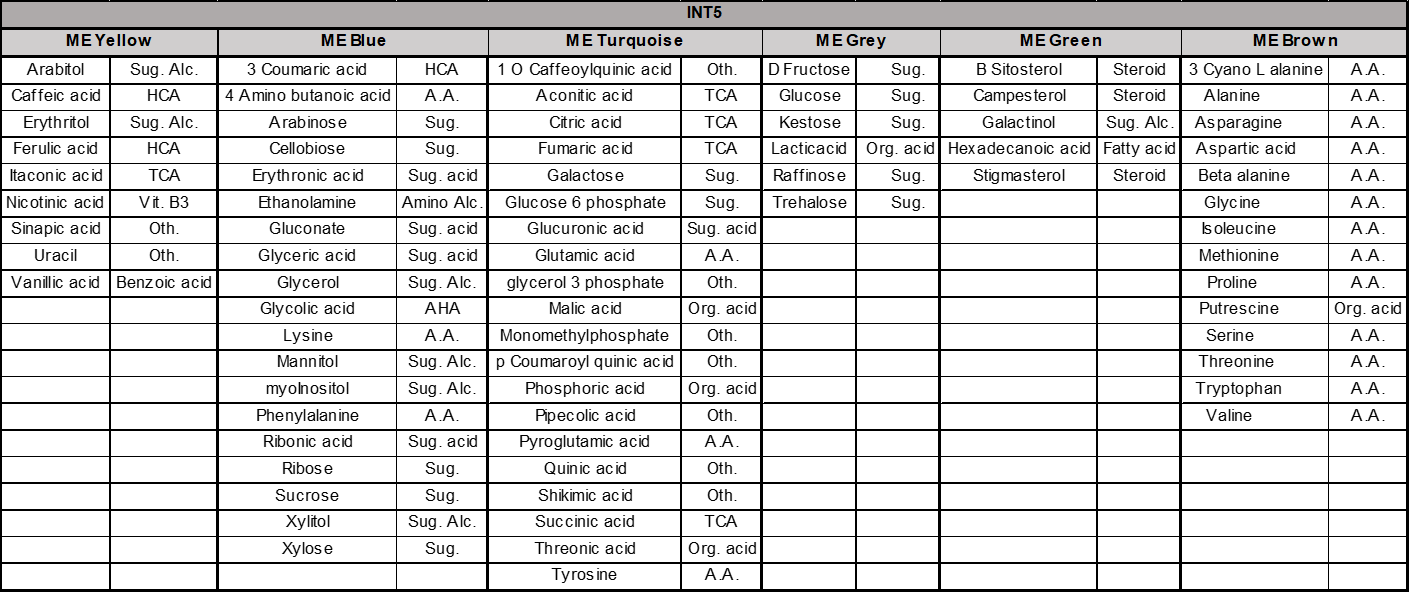


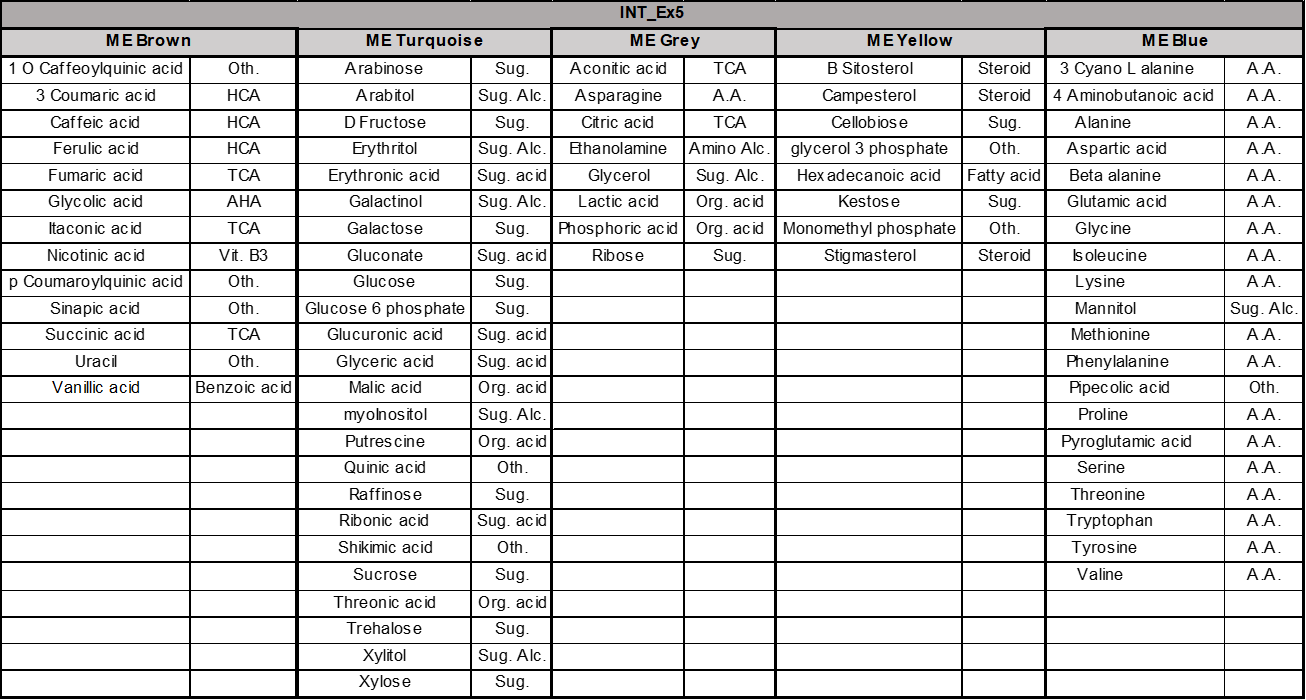


**Table S4.** List of metabolites with highest positive and negative correlation with BRIX in the mid-season for INT5 and INT_Ex5 with P.value > 0.1. Metabolites with positive MS (Metabolite Significance) were displayed in red, and negative in blue.

| **BRIX METABOLITES SIGNIFICANCE (MS)** | | | | |
| --- | --- | --- | --- | --- |
| **STAGE** | **Metabolites** | **Class** | **MS.Brix_Jun_2018** | **p.MS.Brix_Jun_2018** |
| INT5 | D_Fructose | Sugars | 0.294988706 | 0.013173358 |
| INT5 | Glucose | Sugars | 0.290554057 | 0.014684402 |
| INT5 | Kestose | Sugars | 0.218541748 | 0.069130233 |
| INT5 | Alanine | Amino acids | 0.20942128 | 0.08186849 |
| INT5 | Isoleucine | Amino acids | 0.206719268 | 0.085980029 |
| INT5 | Xylitol | Sugar alcohols | 0.205588702 | 0.087748038 |
|  | | | | |
| INT_Ex5 | Monomethylphosphate | Other, MMP | 0.383848296 | 0.000873007 |
| INT_Ex5 | Glutamic_acid | Amino acids | 0.250290944 | 0.033964118 |
| INT_Ex5 | Pyroglutamic_acid | Amino acids | 0.23848438 | 0.043653688 |
| INT_Ex5 | Hexadecanoic_acid | Fatty acid | 0.216050466 | 0.068340155 |
| INT_Ex5 | myo_Inositol | Sugar alcohols | -0.197039294 | 0.097119818 |
| INT_Ex5 | Vanillic_acid | Benzoic acid | -0.21056371 | 0.075834265 |
| INT_Ex5 | Phenylalanine | Amino acids | -0.23244777 | 0.049429399 |
| INT_Ex5 | Galactinol | Sugar alcohols | -0.245812932 | 0.037402926 |
| INT_Ex5 | Quinic_acid | Other, cyclohexanecarboxylic acid | -0.259204756 | 0.027902264 |
| INT_Ex5 | Raffinose | Sugars | -0.260953232 | 0.026827188 |
| INT_Ex5 | Malic_acid | Organic acids | -0.260990896 | 0.02680442 |
| INT_Ex5 | Glyceric_acid | Sugars acid | -0.262057332 | 0.026166506 |
| INT_Ex5 | Erythronic_acid | Sugars acid | -0.297114663 | 0.011261008 |
| INT_Ex5 | Erythritol | Sugar alcohols | -0.329789017 | 0.004669585 |
| INT_Ex5 | Trehalose | Sugars | -0.343335645 | 0.003150801 |
| INT_Ex5 | Arabinose | Sugars | -0.367964406 | 0.001472747 |
| INT_Ex5 | Xylose | Sugars | -0.478699838 | 2.11E-05 |
| INT_Ex5 | Arabitol | Sugar alcohols | -0.482995209 | 1.73E-05 |
| INT_Ex5 | D_Fructose | Sugars | -0.53891871 | 1.04E-06 |
| INT_Ex5 | Xylitol | Sugar alcohols | -0.545983068 | 7.03E-07 |
| INT_Ex5 | Galactose | Sugars | -0.549883228 | 5.64E-07 |
| INT_Ex5 | Glucose | Sugars | -0.552194998 | 4.94E-07 |

**Table S5.** List of metabolites with highest positive and negative correlation with FIBRE in the mid-season for INT5 and INT_Ex5 with P.value > 0.1. Metabolites with positive MS (Metabolite Significance) were displayed in red, and negative in blue.

| **FIBRE METABOLITES SIGNIFICANCE (MS)** | | | | |
| --- | --- | --- | --- | --- |
| **STAGE** | **Metabolites** | **Class** | **MS.Fibre_Jun_2018** | **p.MS.Fibre_Jun_2018** |
| INT5 | Sinapic_acid | Other, phenolic Aldehyde | -0.314698116 | 0.007970291 |
| INT5 | myo_Inositol | Sugar alcohols | -0.268311635 | 0.024717567 |
| INT5 | Serine | Amino acids | -0.244336265 | 0.041503501 |
| INT5 | Glycolic_acid | AHA | -0.227594472 | 0.058111598 |
| INT5 | p_Coumaroyl_quinic_acid | Other, cinnamate ester | -0.221525308 | 0.065327182 |
| INT5 | Glucose | Sugars | -0.213417977 | 0.076074501 |
| INT5 | Mannitol | Sugar alcohols | -0.208967395 | 0.082548035 |
| INT5 | Kestose | Sugars | -0.203067646 | 0.091793758 |
|  | | | | |
| INT_Ex5 | Galactinol | Sugar alcohols | 0.333919837 | 0.004149241 |
| INT_Ex5 | Malic_acid | Organic acids | 0.306038483 | 0.008937725 |
| INT_Ex5 | Pyroglutamic_acid | Amino acids | 0.296627516 | 0.011401704 |
| INT_Ex5 | Raffinose | Sugars | 0.22782193 | 0.054267719 |
| INT_Ex5 | Xylitol | Sugar alcohols | -0.202187509 | 0.088524701 |
| INT_Ex5 | Threonine | Amino acids | -0.207128738 | 0.080850508 |
| INT_Ex5 | Erythritol | Sugar alcohols | -0.227157411 | 0.054993491 |
| INT_Ex5 | Mannitol | Sugar alcohols | -0.242492549 | 0.040135899 |
| INT_Ex5 | Kestose | Sugars | -0.243156557 | 0.039576491 |
| INT_Ex5 | Beta_alanine | Amino acids | -0.243481191 | 0.039305361 |
| INT_Ex5 | 4_Aminobutanoic_acid | Amino acids | -0.252266594 | 0.032533283 |
| INT_Ex5 | Glycolic_acid | AHA | -0.252691499 | 0.032232245 |
| INT_Ex5 | Valine | Amino acids | -0.262065678 | 0.026161564 |
| INT_Ex5 | Serine | Amino acids | -0.293759323 | 0.012261431 |
| INT_Ex5 | Proline | Amino acids | -0.303189646 | 0.009629141 |
| INT_Ex5 | Tyrosine | Amino acids | -0.314247632 | 0.007182097 |
| INT_Ex5 | Tryptophan | Amino acids | -0.324602631 | 0.005404208 |
| INT_Ex5 | Alanine | Amino acids | -0.326196751 | 0.005168237 |
| INT_Ex5 | Isoleucine | Amino acids | -0.32922898 | 0.004744406 |
| INT_Ex5 | Pipecolic_acid | Other, Alkaloids | -0.330789672 | 0.004538497 |
| INT_Ex5 | Arabitol | Sugar alcohols | -0.400696484 | 0.000486867 |
| INT_Ex5 | Phenylalanine | Amino acids | -0.411260649 | 0.000332236 |

**Table S6.** Top pathway enrichment of metabolites for INT5 and INT_Ex5, for each of the metabolite Eigengenes Modules (ME), labelled by their colours. Ranking ordered by P value, Holm and false discovery rate (FDR).


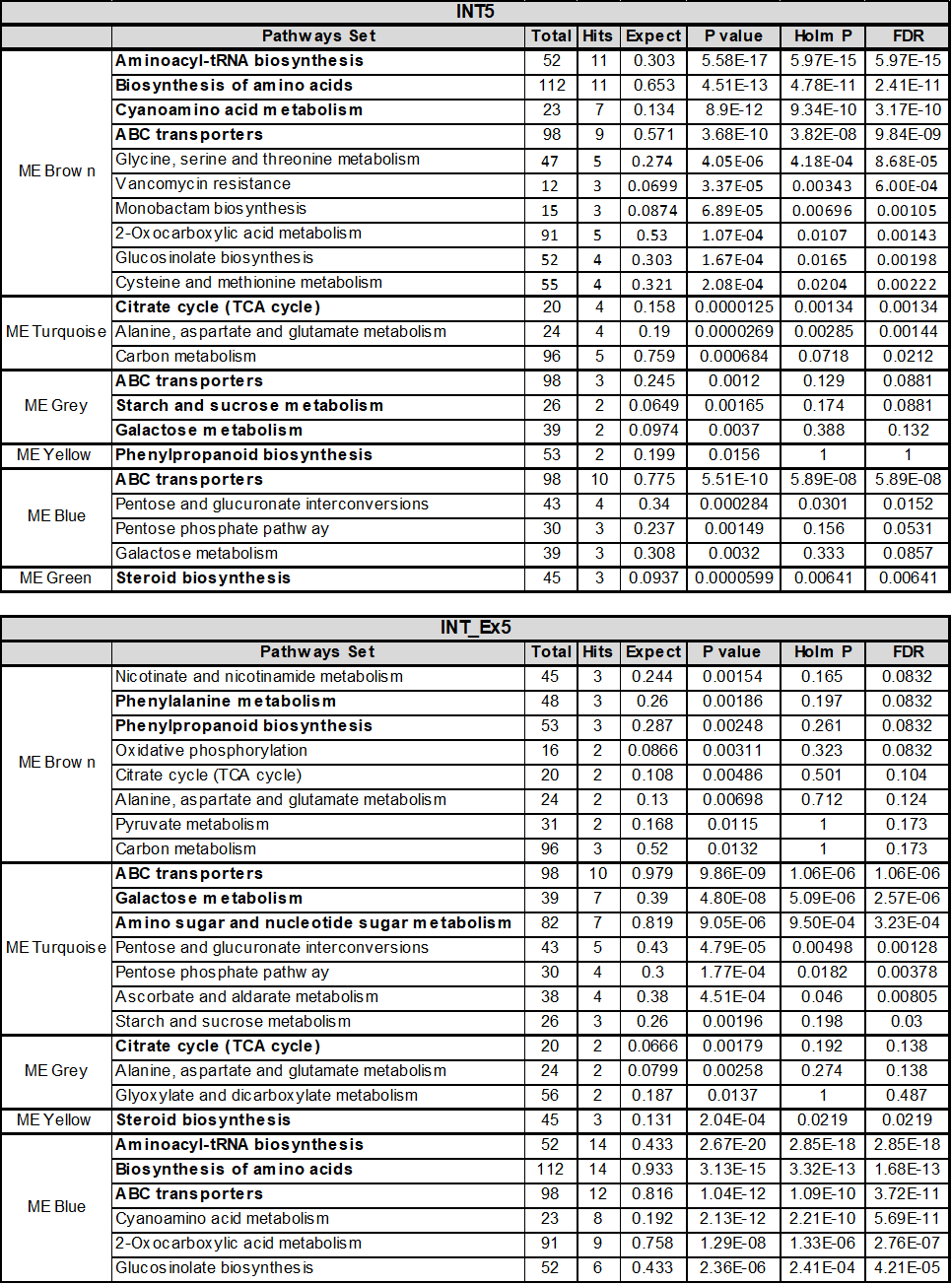


| **A** | |
| --- | --- |
| 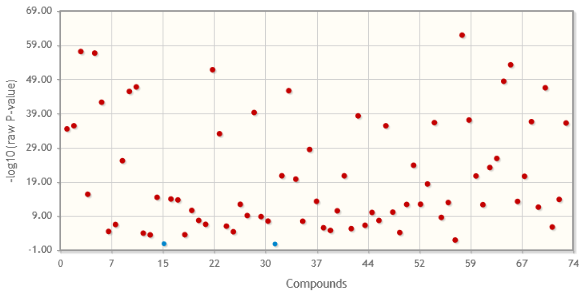 | |
| **B** | **C** |
| 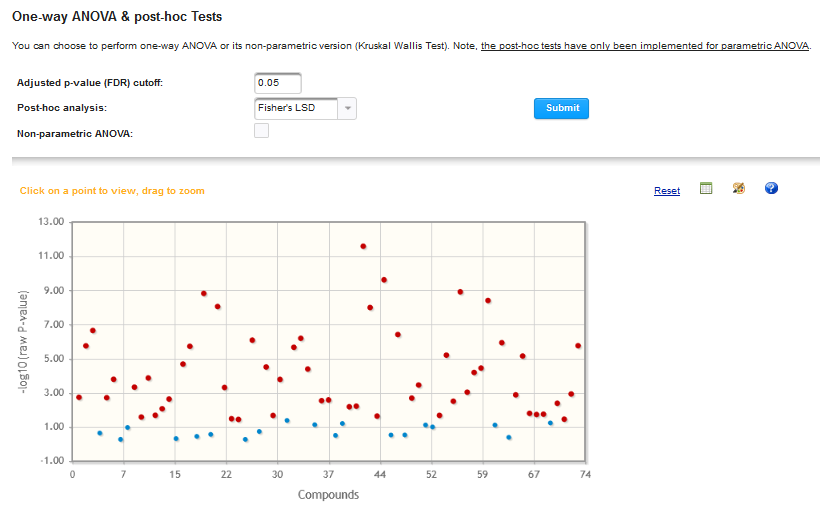 | 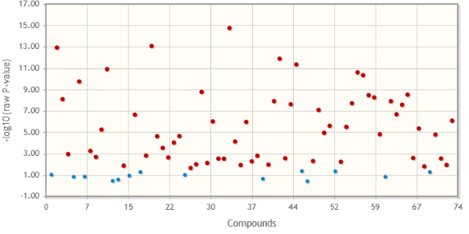 |

**Figure S1.** One-way ANOVA analysis of the 74 metabolites among the 24 genotypes **(A)** between internodes of the 5 stages **(B)** for internode INT5 **(C)** for internode INT_Ex5. Significant metabolites were displayed in red and non-significant metabolites in blue. Fisher’s LSD post-hoc analysis p.value cutoff < 0.05.

| **A**  D-Fructose - INT5 | **B**  Glucose - INT5 | **C**  Sucrose - INT5 |
| --- | --- | --- |
| 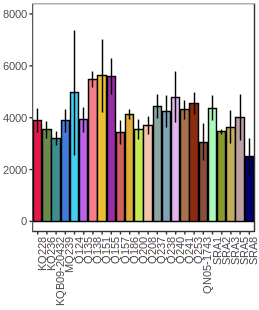 | 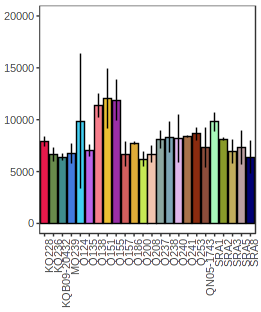 | 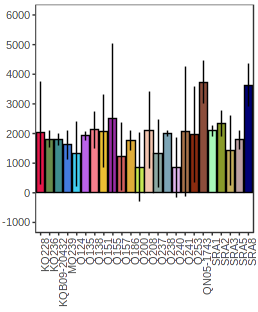 |
| **D**  D-Fructose - INT_Ex5 | **E**  Glucose - INT_Ex5 | **F**  Sucrose - INT_Ex5 |
| 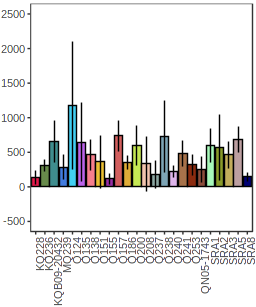 | 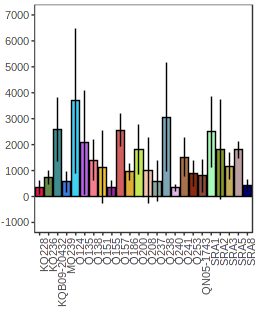 | 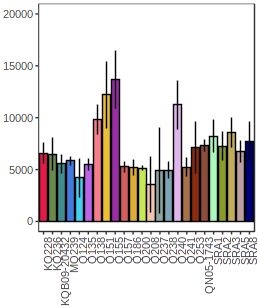 |

Figure S2. Details view from one-way ANOVA analysis of three sugars. X-axis shows the 24 genotypes and y-axis shows the original relative concentration, using MetaboAnalyst 4.0 for (A) D-Fructose for INT5, p.value=1.2E-4 (B) Glucose for INT5, p.value=4.85E-5 (C) Sucrose for INT5, p.value=2.75E-4 (E) D-Fructose for INT_Ex5, p.value=2.18E-4 (F) Glucose for INT_Ex5, p.value=0.003 (G) Sucrose for INT_Ex5, p.value=2.7473E-13.

| **INT_5** |
| --- |
| 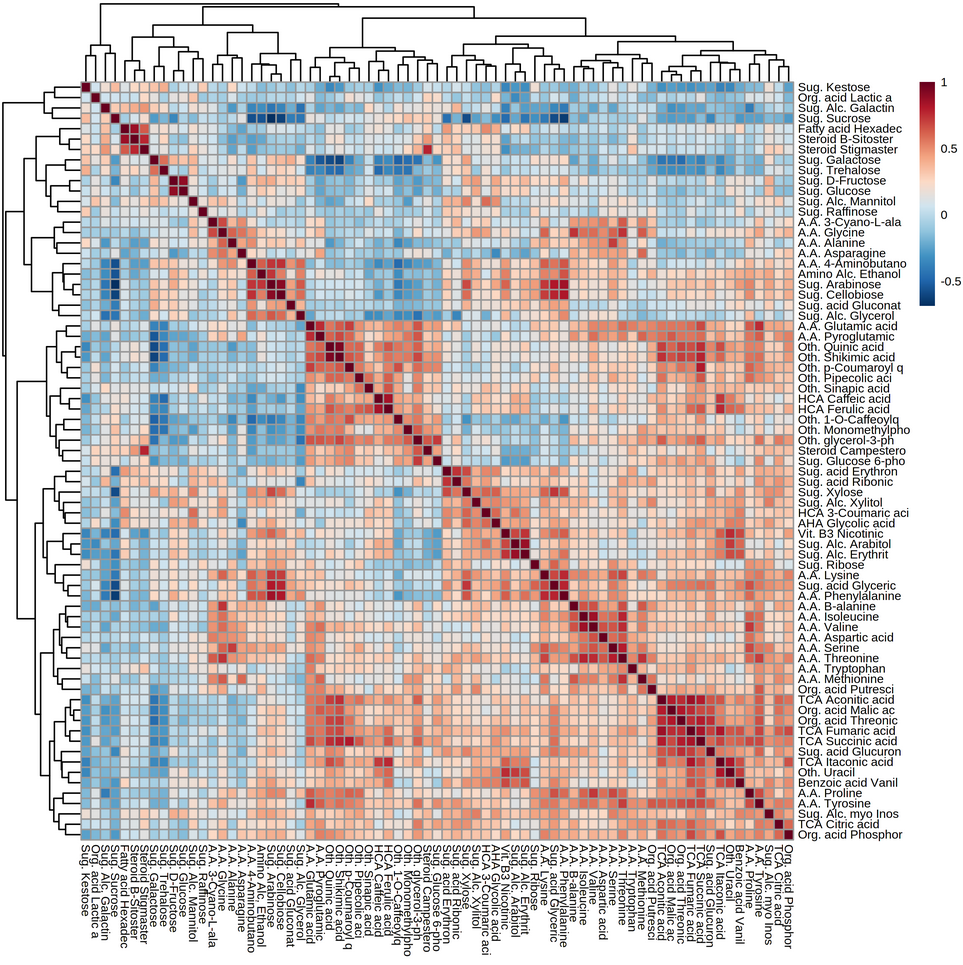 |
| **INT_Ex5** |
| 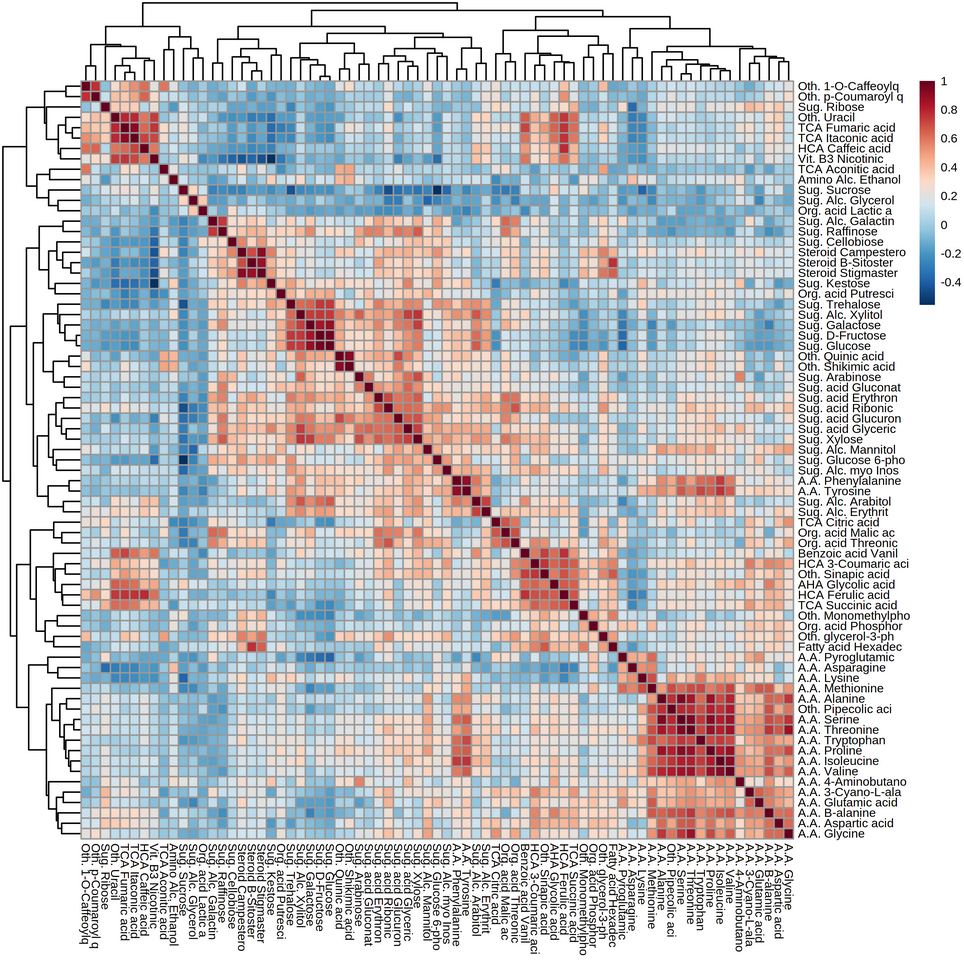 |

**Figure S3.** Heatmap of Pearson correlation heatmap of metabolites. On the top Internode INT5, bottom INT_Ex5. Positive correlations were represented in red and negative in blue.


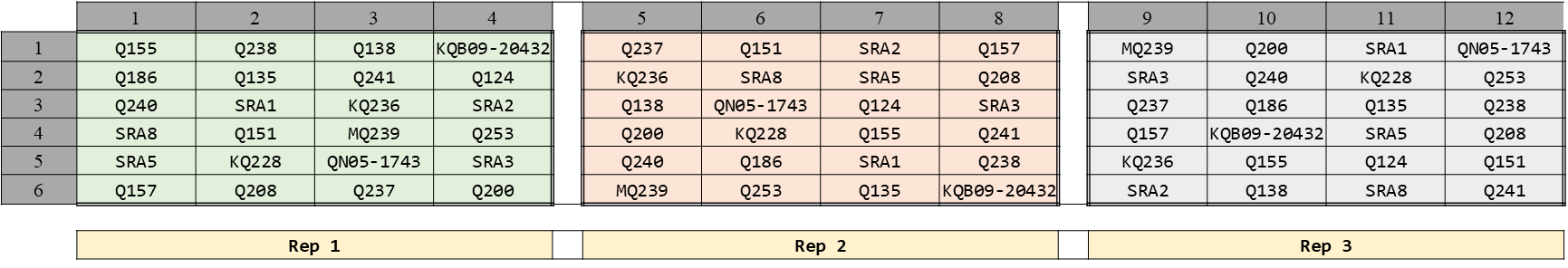
  **Figure S4.** Field map of the 24 genotypes in 3 replicates.
